# Factors associated with Khat use among youths visiting HIV testing and counseling centers in Gamo Gofa, Southern Ethiopia

**DOI:** 10.1186/1471-2458-13-1199

**Published:** 2013-12-18

**Authors:** Marelign Tilahun, Gistane Ayele

**Affiliations:** 1 Department of Public Health, College of Medicine and Health Sciences, Arba-Minch University, P.O. Box: 21, Arba-Minch, Ethiopia

## Abstract

**Background:**

The use of khat among youths can be harmful leading to decreased academic performance, increased risk of contracting HIV and other sexually transmitted diseases or other psychiatric symptoms. It is believed to be one of the factors associated with unprotected risky sexual behavior predisposing the youth for HIV infection and transmission.

**Methods:**

A cross-sectional study was conducted in South West Ethiopia. A total of 410 participants were recruited in the study using stratified sampling technique. Data were collected by using interviewer administered structured questionnaire. Multiple logistic regression and Cox regression were used to assess the association of independent variables with the outcome variable.

**Result:**

Khat use was positively associated with male gender (OR 2.9; C.I. 1.4 to 6.0), alcohol use (OR 4.8; C.I. 2.1 to 10.6), no education level (OR 2.6; C.I. 1.1 to 6.2) and not having communication with parents about khat chewing (OR 2.6; C.I. 1.1 to 6.2).

**Conclusion:**

Strategies should be designed to increase awareness of factors associated with khat use among youths and their parents in order to reduce the prevalence of khat use and its adverse social and health consequences.

## Background

Khat is a strong stimulant that causes mild to moderate psychological dependence although not as strong as that of alcohol and tobacco and its consumption can have serious health and economic consequences. Its effects on the chewer include increased levels of energy, increased self-esteem, euphoria, increased libido, excitement, and increased proclivity for social interaction [1,2]. Khat is widely consumed among the youths of Ethiopia as shown by several prevalence studies [3,4]. According to the Ethiopian Demographic and Health Survey report, the point prevalence of khat chewing among men and women during 2011 is 28% and 11% respectively. Among both women and men, khat use increases with age. Among women, khat consumption is higher in rural areas than in urban areas (12 percent versus 7 percent) while among men there is no marked difference by place of residence [5].

The use of khat among youths can be harmful, leading to decreased academic performance, increased risk of contracting HIV and other sexually transmitted diseases or other psychiatric symptoms such as lethargy, hopelessness and insomnia [6]. Insomnia is a common problem associated with the use of khat which prompts the chewer to use/abuse sedatives and to indulge in alcohol as a means of overcoming the side effect and this is believed to be one of the factors associated with unprotected risky sexual behavior predisposing the youth for HIV infection and transmission [1,7,8]. Even though khat chewing have become common practice among youths in Ethiopia, only very few studies have assessed its magnitude and the associated factors [7,8]. As such studies have not been conducted in South-West Ethiopia; we assessed the prevalence of khat use and contributing risk factors in this area.

## Methods

A cross-sectional study was conducted from January 15 – March 20, 2012. The study was conducted in Gamo-Gofa zone which is located about 505 km south west from Addis Ababa, about 275 km from Awassa, the capital of the southern nations, nationalities and peoples region. According to the 2007 census result it has a population of 1,595,570 and of this 794,485 were males and 801,085 were females. The study population was all youths attending HIV testing and counseling centers in public health facilities during the data collection period.

### Sampling procedure

Young subjects (15 to 24 years of age) were recruited by stratified sampling technique using clients of three hospitals and health centers offering HIV testing and counseling services.

### Inclusion and exclusion criteria

Youths attending HIV testing and counseling centers were included in the study and those having complete hearing problem and unable to communicate with sign language were excluded.

### Sample size determination

A total sample size of 410 were determined by using the formula of single population proportion estimation ( n = Zα/2^2^ p(1-p) / w^2^) with the following parameters; proportion of khat use 41% which is taken from a previous study conducted in other parts of Ethiopia [1], 5% margin of error, 95% confidence interval and by adding 10% non-response.

### Data collection

Data were collected about khat use (yes/no), last year use, last month use, and a variety of socio-demographic variables, including income and profession.

### Operational definition

A study participant was considered as ever having been a chewer if he/she responds yes to the question ‘Have you ever chewed khat in your life?’ Then follow up questions were employed to collect information such as khat chewing in the past one year. Current khat use is defined as use of khat at least once during the past 30 days before the survey and these operational definitions were adopted from the Ethiopian demographic and health survey 2011.

### Data processing and analysis

Data were entered and analyzed using SPSS software version 16. Descriptive statistics such as frequencies and proportion was used to describe the study population in relation to relevant variables. Multivariate logistic regression was used to assess the presence and degree of association between dependent and independent variables.

### Ethical consideration

Ethical clearance was obtained from ethical review board of Arba-Minch University and permission to conduct the study in each health facility was secured from the respective health institutions. Verbal informed consent was obtained from each study participant and written consent from the parents or legal guardians of participants less than 18 years.

## Results

Out of 410 youths recruited, 400 (97.6%) were included. Ninety seven (24.2%) respondents were married, 273 (68.3%) not married, and the rest was divorced or widowed) (Table 1).

**Table 1:** Socio-demographic characteristics of youths visiting HIV testing and counseling centers in Gamo-Gofa, South West Ethiopia, 2012

**Variables**	**Khat chewers: n (%)**	**Non khat chewers: n (%)**	**Total**
**Age** [Mean (±SD) = 20.57 (±2.59)]			
15 - 19	17 (4.2%)	92 (23.0%)	109 (27.2%)
20 - 24	91 (22.8%)	200 (50.0%)	291 (72.8%)
**Sex**			
Male	68 (17.0%)	117 (29.2%)	185 (46.2%)
Female	40 (10.0%)	175 (43.8%)	215 (53.8%)
**Residence**			
Urban	90 (22.5%)	208 (52.0%)	298 (74.5%)
Rural	18 (4.5%)	84 (21.0%)	102 (25.5%)
**Religion**			
Orthodox Christian	80 (20.0%)	139 (34.8%)	219 (54.8%)
Muslim	6 (1.5%)	11 (2.8%)	17 (4.2%)
Protestant	22 (5.5%)	134 (33.5%)	156 (39.0%)
Others*	2 (0.5%)	6(1.5%)	8 (2.0%)
**Education**			
No education	8 (2.0%)	250 (12.4%)	58 (14.4%)
Primary education	13 (3.3%)	34 (8.5%)	47 (11.8%)
Secondary & above	87 (21.8%)	208 (52.0%)	295 (73.8%)
**Occupation**			
Government employed	26 (6.5%)	46 (11.5%)	72 (18.0%)
Merchant	11 (2.8%)	37 (9.2%)	48 (12.0%)
House wife	7 (1.8%)	24 (6.0%)	31 (7.8%)
Daily laborer	18 (4.4%)	36 (9.0%)	54 (13.4%)
Student	46 (11.5%)	149 (37.3%)	195 (48.8%)
**Marital status**			
Never married	85 (21.3%)	188 (47.0%)	273 (68.3%)
Married/living together	15 (3.8%)	82 (20.4%)	97 (24.2%)
Divorced/separated/widowed	8 (2.0%)	22 (5.5%)	30 (7.5%)
**Monthly income**			
<= 450 Ethiopian currency ( <= $ 25 )	58 (14.5%)	182 (45.5%)	240 (60.0%)
451 – 999 Ethiopian currency ( $ 25.1 - $ 55.5 )	14 (3.5%)	57 (14.3%)	71 (17.8%)
> = 1000 Ethiopian currency ( > = $ 55.6 )	36 (9.0%)	53 (13.2%)	89 (22.2%)

* Catholic, Adventist, Only Jesus.

### Differences of the study participants with respect to age at initiation of khat use

As shown in Table 2, females were found to initiate khat use at earlier age than male respondents (RR 2.0; C.I. 1.4 to 2.8). With respect to occupation, daily laborers were found to initiate khat use at earlier age (RR 1.9; C.I. 1.2 to 3.1). Those who live alone were found to initiate khat use at earlier age compared to those who live with their spouse (RR 3.1; C.I. 1.5 to 6.1). Those respondents who ever had used alcohol were also found to initiate khat use at earlier age than those who didn’t use alcohol (RR 2.3; C.I. 1.6 to 3.4).

**Table 2:** Differences of the study participants with respect to age at initiation of khat use in south west Ethiopia, 2012

**Ever used khat before 18 years**	**RR (95% CI)**
**Sex**	
Male	1.0
Female	2.0 (1.4, 2.8)
**Occupation**	
Government employed	1.0
Merchant	1.1 (0.5, 2.1)
House wife	0.9 (0.4, 2.2)
Daily Laborer	1.9 (1.2, 3.1)
Student	1.3 (0.7, 2.4)
**Alcohol use**	
Yes	2.3 (1.6, 3.4)
No	1.0
	1.00
**Live with**	
Spouse	1.0
Parents	3.1 (1.5, 6.1)
Alone	3.4 (1.8, 6.7)

### Factors associated with khat use among youths in Gamo-Gofa, South West Ethiopia

The multivariate logistic regression analysis in Table 2 showed that the odds of khat use were almost three times higher among males compared to females (OR 2.9; C.I. 1.4 to 6.0). Alcohol users were 4.8 times (OR 4.8; C.I. 2.1 to 10.6) more likely to use khat than their counterparts. Compared to youths who do not have communication with their parents about STI and HIV, those who do have were 2.2 times [OR 2.2; C.I. 1.1 to 4.1) more likely to use khat and youths who do not have communication with their parents about khat chewing were 2.9 times [OR 2.9; C.I. 1.3 to 6.5) more like to use khat than their counterparts Table 3.

**Table 3:** Logistic regression model estimates of risk factors for khat chewing among youths (Crude & adjusted OR) in Gamo-Gofa, South West Ethiopia, 2012

**Explanatory variable**	**Khat use**		**Crude OR (95% CI)**	**Adjusted OR (95% CI)**	**P-value**
	**Yes (1)**	**No (0)**			
**Sex**					
Male	84	110	2.5 (1.5, 4.4)	2.9 (1.4, 5.9)	0.004
Female	50	161	1.0	1.0	
**Alcohol use**					
Yes	110	101	5.1 (3.6, 14.8)	4.7 (2.1, 10.6)	<0.001
No	34	160	1.0	1.0	
			1.00		
**Education level**					
No education	8	51	2.6 (1.2, 5.7)	2.6 (1.1, 6.2)	0.047
Primary education	13	34	1.1 (0.5, 2.1)	0.9 (0.4, 1.9)	
Secondary & above	87	212	1.0	1.0	
**Occupation**					0.026
Government employed	26	47	0.6 (0.3, 0.9)	1.8 (0.7, 4.4)	
Merchant	11	39	1.1 (0.5, 2.3)	2.2 (1.7, 6.4)	
House wife	7	24	1.1 (0.4, 2.6)	0.9 (0.4, 2.1)	
Daily laborer	18	36	0.6 (0.3, 1.2)	2.1 (1.1, 3.9)	
Student	46	151	1.0	1.0	
			1.00		
**Monthly income**					
<= 450 Ethiopian currency ( <= $ 25)	36	54	1.0	1.0	0.004
451 – 999 Ethiopian currency ( $ 25.1 - $ 55.5)	58	185	2.1 (1.3, 3.6)	2.4 (1.4, 4.1)	
> = 1000 Ethiopian currency ( > = $ 55.6)	14	58	2.8 (1.3, 6.7)	2.7 (1.3, 5.8)	
**Have communication with parents about STI and HIV**					0.017
Yes	52	187	1.0	1.0	
No	55	104	1.9 (1.2, 3.0)	2.2 (1.1, 4.1)	
**Have communication with parents about khat chewing**					
Yes	8	57	1.0	1.0	0.008
No	63	167	2.7 (1.2, 6.0)	2.9 (1.3, 6.5)	

## Discussion

The findings of this study reveal the prevalence of khat chewing among youths to be 27.0% which is comparable to the one that is reported for Jimma University (26.7%). This could be due to the fact that both studies were conducted in the neighboring locality [9].Generally; the prevalence of khat chewing in this study seems to be higher than studies carried out in other parts of the country. A study from north-western Ethiopia [10] revealed that the prevalence of khat chewing among college students to be 17.5%. Another study carried out in Addis Ababa, Ethiopia found a prevalence of khat chewing among university students to be 14.0% [6], where as a survey of 1,890 secondary school students from eastern Ethiopia reported prevalence of 24.2% [4]. These figures are lower than what we found. This may be because of the fact that our study included samples of youths who come from rural communities who are directly economically dependent on khat through cultivation and marketing it. This may mean that they are also likely to develop the habit of consuming khat. Or the difference might be because of availability of income or youths may be encouraged to ‘try’ khat chewing by parents and neighbors as evidenced by a study from Yemen in which school-aged children as young as eight years are provided with khat with the belief that they could study better and become energetic [2]. In neighboring Somalia about 36.4% of respondents reported use of khat [11] and this might be due to the fact that the Somali sample is composed of combatants reportedly under severe stress and in a context of social disruption which potentially increases substance use, hence the difference with our study.

Youths who are alcohol users (OR 4.7; C.I. 2.1 to 10.6) were found to have higher odds of chewing khat than non-users of alcohol in this study which contrasts with previous study in Butajira, southern Ethiopia where alcohol drinking was significantly (P < 0.001) more common among non-chewers (30.9%) than Khat chewers (7.8%) [12]. This might be attributable to the predominantly Muslim religious background of the study population conducted in Butajira where alcohol consumption is prohibited. Studies conducted in other parts of Ethiopia reveal similar finding where alcohol users were 3.57 times more likely to chew khat than non-users of alcohol in Addis Ababa, Ethiopia [6]. The higher odds of alcohol use among khat chewers might be due to the fact that the side effects of khat may prompt the chewers to indulge in alcohol to counteract the stimulant properties and facilitate sleep.

Male youths (OR 2.9; C.I. 1.4 to 5.9) were found to have higher odds of chewing khat than females in this study. This is similar to a study conducted in Gondar among college students [10], where males were found to be 3.69 times more likely to use khat compared with females. Other studies conducted in Addis Ababa, Ethiopia and Harar, eastern Ethiopia also showed a similar finding where males were found to be 2.35 and 2.1 times more likely to use khat than females in Addis Ababa and Harar respectively [2, 8,].A similar finding has also been reported among secondary school students in Saudi Arabia showing significant differences in chewing between males and females [13]. This might be due to the common tendency of males to abuse substances compared to females and to the greater cultural acceptance of male substance use in Ethiopia and among other khat consuming countries.

Initiation of substance use before 18 years of age may increase a potential exposure to HIV/AIDS by causing loss of inhibition and involvement in risky sexual behaviors such as early sexual initiation, unprotected sex, multiple sexual partners, prolonged and traumatic sex, and risky injections [14-16]. In this study the median (IQR) age for starting chewing khat was 16.4 (3.0) years. The median age for initiation of khat use was reported to be 17.3 years among college students in a study from northwestern Ethiopia [10], 15.1 years among high school students from eastern Ethiopia [4]. This shows that the participants in our study area start the habit earlier than what have been reported from the northern Ethiopia. This could be explained by the fact that the cultivation and consumption of khat is practiced widely in the southern Ethiopia and it is more a part of the culture of the study area than that of the northern regions.

### Limitation

Social desirability bias is a potential limitation of this study. Another limitation is the use of cross sectional data and this makes it difficult to establish causality. The study is also health facility based and therefore precludes generalization to all youths in Ethiopia. Despite this limitation, the study provides useful information that will inform health service planners to design a strategy to reduce the prevalence of khat chewing habit and its adverse social and health consequences.

## Conclusion

In conclusion, khat chewing is prevalent among youths in the study area and it is independently associated with male gender, alcohol use, being daily laborer, no education, not having communication with parents about sexually transmitted infections and HIV and about the risks of khat use. Strategies should be designed to increase awareness of factors associated with khat use among youths and their parents in order to reduce the prevalence of khat use and its adverse social and health consequences.

## Competing interests

The authors declare that they have no competing interests.

## Authors’ contributions

MT was investigator, involved in proposal writing, designing, and recruitment and training of supervisors and data collectors, analysis and write-up and in all stages of the project implementation. He did most of the analysis and write up of the paper. GA contributed in the designing of the methodology, recruitment and training of supervisors and data collectors and involved in designing of project proposal, design of questionnaires, supervision and involved in the final approval of the paper. Both authors read and approved the final manuscript.

## Pre-publication history

The pre-publication history for this paper can be accessed here:

http://www.biomedcentral.com/1471-2458/13/1199/prepub

## References

[B1] Dawit A Asfaw D Amare D Khat chewing habit as a possible risk behavior for HIV infection Ethiop J Health Dev 2005 13 3 174 181

[B2] Almotareb A Baker K Broadley K Khat: pharmacological and medical aspects and its social use in Yemen, a review article Phytother Res 2002 13 403 413 10.1002/ptr.1106 12203257

[B3] Derege K Atalay A Getnet M Khat and alcohol use and risky sex behavior among in-school and out-of-school youth in Ethiopia BMC Public Health 2005 13 109 10.1186/1471-2458-5-109 16225665 PMC1274331

[B4] Ayalu A Asmamaw M Sibhatu B Berhanu Y Prevalence and determinants of Khat (Catha edulis) chewing among high school students in eastern Ethiopia: a cross- sectional study PLoS One 2012 13 3 e33946 10.1371/journal.pone.0033946 22479484 PMC3316517

[B5] Central Statistical Agency [Ethiopia] and ORC Macro Ethiopia Demographic and Health Survey 2011. Addis Ababa, Ethiopia and Calverton, Maryland, USA 2012 Ethiopia: Central Statistical Agency and ORC Macro

[B6] Wakgari D Aklilu A Substance use and its predictors among undergraduate medical students of Addis Ababa University in Ethiopia BMC Public Health 2011 13 660 10.1186/1471-2458-11-660 21859483 PMC3170623

[B7] Belew M Kebede D Kassaye M Enquoselassie F The magnitude of khat use and its association with health, nutrition and socio-economic status Ethiop Med J 2000 13 1 11 26 11144876

[B8] Seme A Mariam D Worku A The association between substance abuse and HIV infection among people visiting HIV counseling and testing centers in Addis Ababa, Ethiopia Ethiop J Health Dev 2005 13 116 125

[B9] Gelaw Y Haileamlak A Khat chewing and its socio-demographic correlates among the staff of Jimma University Ethiop J Health Dev 2004 13 3 179 184

[B10] Kebede Y Cigarette smoking and khat chewing among college students in Northwest Ethiopia Ethiop J Health Dev 2002 13 1 9 17

[B11] Odenwald M Hinkel H Schauer E Neuner F Schauer M The Consumption of Khat and other drugs in Somali Combatants: a cross- sectional study PLoS Med 2007 e341 18076280 10.1371/journal.pmed.0040341 PMC2121109

[B12] Getahun W Gedif T Tesfaye F Regular Khat (*Catha edulis*) chewing is associated with elevated diastolic blood pressure among adults in Butajira, Ethiopia: a comparative study BMC Public Health 2010 13 390 10.1186/1471-2458-10-390 20594361 PMC2912809

[B13] Ageely H Prevalence of Khat chewing in college and secondary (high) school students of Jazan region, Saudi Arabia Harm Reduct J 2009 13 1 11 10.1186/1477-7517-6-1 19545389 PMC2707373

[B14] Federal HIV/AIDS Prevention and Control Office, Federal Ministry of Health Single point HIV prevalence estimate 2007 Addis Ababa, Ethiopia: Federal Ministry of Health 8 17

[B15] Rehm J Room R Monteiro M Alcohol as a risk factor for global burden of disease Eur Addict Res 2003 13 157 164 10.1159/000072222 12970584

[B16] Paul C Angela O Interventions to reduce HIV sexual transmission within discordant couples 2006 BMJ Publishing http://www.scribd.com/doc/78829447/9. Accessed January 08, 2012

